# Transferrin-navigation Nano Artificial Antibody Fluorescence Recognition of Circulating Tumor Cells

**DOI:** 10.1038/s41598-017-10486-9

**Published:** 2017-08-31

**Authors:** Wei Zhang, Jiaoyang Wang, Ping Li, Chuanchen Wu, Hongyan Zhang, Wen Zhang, Hui Wang, Bo Tang

**Affiliations:** 1 College of Chemistry, Chemical Engineering and Materials Science, Collaborative Innovation Center of Functionalized Probes for Chemical Imaging in Universities of Shandong, Key Laboratory of Molecular and Nano Probes, Ministry of Education, Institute of Biomedical Sciences, Jinan, 250014 P.R. China; 2 College of Life Science, Shandong Normal University, Jinan, 250014 P.R. China

## Abstract

Specific recognition of circulating tumor cells (CTCs) is of great significance for cancer diagnosis and personalized therapy. The antibodies and aptamer are commonly used for recognition of CTCs, but they often suffer from low stability and high cost. Therefore, chemically stable and low-cost artificial recognition elements are still highly demanded. Herein, we prepared nano artificial antibody based on molecular imprinting and applied for fluorescence recognition of CTCs. Surface imprinting was employed to construct a transferrin (TRA)-imprinted layer on the surface of rhodamine doped silica nanoparticles. Take advantage of the specific interaction between TRA and TRA receptor (overexpressed on cancer cells), the as-prepared TRA-imprinted artificial antibody was allowed for specific targeting cancer cells mediated by TRA. And the average recognition efficiency of the artificial antibody for the cancer cells was 88% through flow cytometry. Finally, the nano artificial antibody was successfully applied to specific identify mimetic CTCs, under the same conditions, the recognition ability of artificial antibody for CTCs was 8 times higher than the white blood cells.

## Introduction

The circulating tumor cells (CTCs) are an important biomarker in vasculature of cancer patients, and have been confirmed to contribute to the formation of metastases in model systems^1, 2^. Currently, biometric unit including antibody, aptamer, and e-selectin were commonly used to identify CTCs, such as microfluidic-based devices and immunomagnetic approaches^3–11^. Although biological recognition unit has good recognition ability, they often suffer from high-cost and easily-denatured^12–17^. Furthermore, some antibodies are difficult to obtain through biological means. Therefore, to develop artificial antibody based on molecular imprinting technique is of great significance.

Molecularly imprinted polymers (MIP) have been recognized as artificial antibody with predesigned binding specificity and affinity toward to template molecules^18, 19^. Compared with natural antibodies, the merits of MIPs are preparation simple and low-cost. Combining nanoparticles and molecular imprinting into an integrated system applied for biosensing and biometrics has received considerable attention^20–31^. Among them, fluorescence MIP combined the merits of high selectivity of MIP and high sensitivity of fluorescence detection, which exhibited potential applications in the field of biological detection^32–38^. Notably, imprinting of small molecules has been well achieved, but protein imprinting still presents difficulties, which is mainly due to the complexity of the protein structure and the variety of their sequence. Especially, a large number of proteins are expressed on the surface of cells, and those proteins play an important role for cell recognition. Therefore, to develop new types of artificial antibodies for cell recognition using protein as a template molecule is of great significance. As transmembrane glycoprotein, transferrin receptor (TRAR) is closely associated with iron transport in living cells, which highly expressed in a variety of cancers cells, about 100-fold more than that on normal cells because cancer cells need more iron to maintain cellular survival^39, 40^. Thus, specific recognition of TRAR is of great importance for cancer diagnosis and therapy. Currently, transferrin (TRA) has proven to be an efficient specific recognition site targeting overexpressed TRAR on cancer cell^41–44^. It should be pointed out that boronate affinity play important roles for recognition of the cis-diol containing structures^45^. Thus, combining merits of MIP and boronate affinity was able to develop new recognition element, the resultant functional materials could achieve higher specific binding ability for TRA through synergistic effect. Therefore, to develop TRA-imprinted fluorescence artificial antibody is good choice for imaging of TRAR on cell surface mediated by TRA. However, further exploration is still needed. More importantly, it is critical to prove whether the approach is applicable for specific recognition of TRAR on cancer cells surface. If the design route is feasible, to develop TRA-based imprinted artificial antibody is highly desirable.

**Figure 1:**
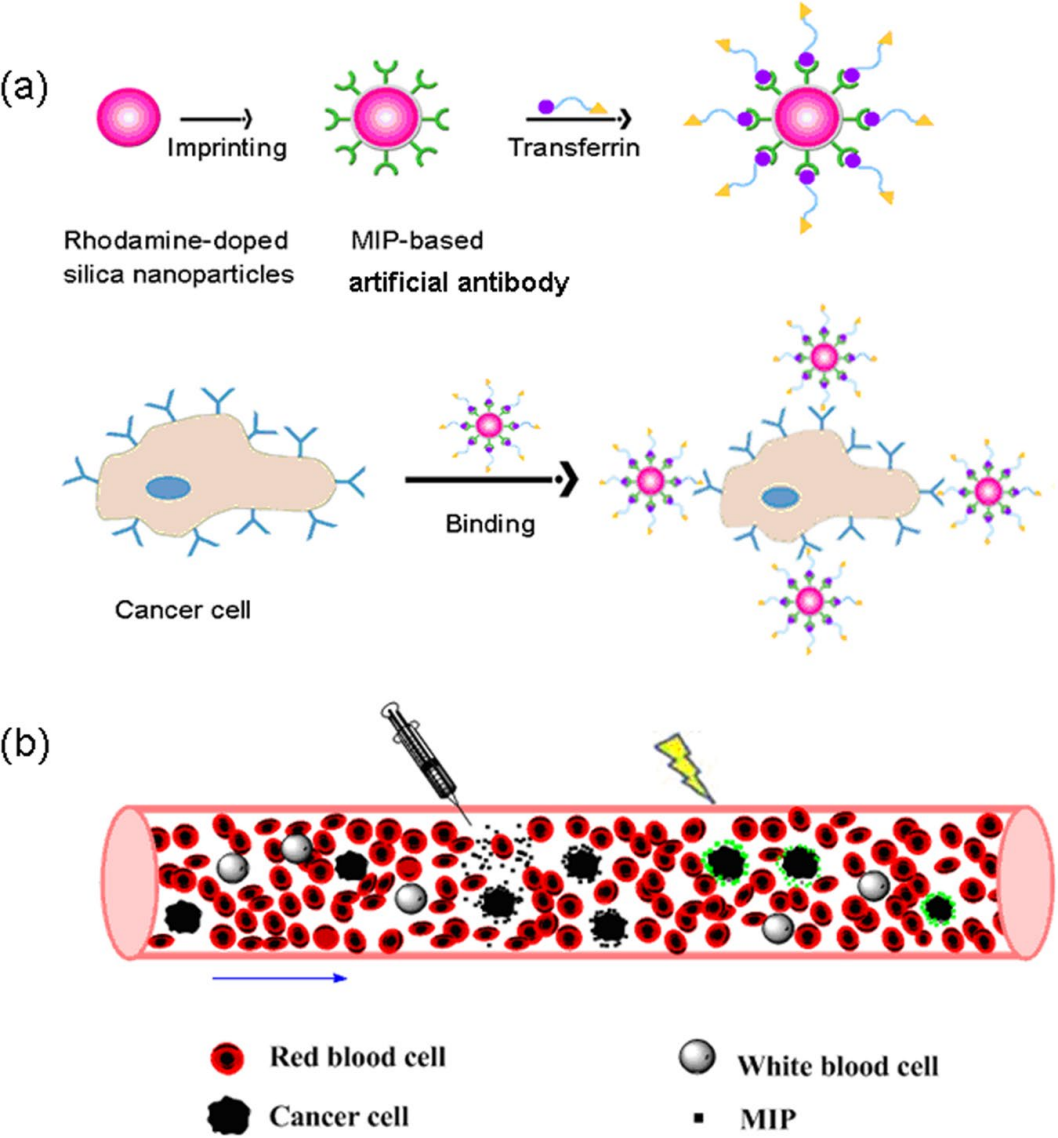
(**a**) Schematic of the synthesis route of Rhodamine-doped silica nanoparticles, TRA-imprinted Rhodamine-doped silica artificial antibody, and the scheme to illustrate the interaction between TRA-imprinted artificial antibody and cancer cells; (**b**) Schematic of the application of the artificial antibody for CTCs.

In this work, we demonstrate that cancer cell recognition can be achieved via TRA-imprinted artificial antibody, and the as-prepared artificial antibody was applied for targeting and fluorescence recognition of TRAR in cancer cells. The synthesis route and recognition principle of the artificial antibody for TRAR in cancer cells is illustrated in Fig. 1. The glycoprotein TRA was selected as the templates. Surface imprinting approach was used to make the imprinted recognition site generated on the surface of the nanoparticles. In addition, the generality of the method was demonstrated by successful imprinting of another glycoprotein horseradish peroxidase (HRP). Rhodamine doped silica nanoparticles were used as fluorescent materials, and then produced a TRA-imprinted silica layer through surface imprinting. The obtained TRA-imprinted artificial antibody was able to specific recognition of target TRA, which further exhibited the ability to differentiate between cancer cells and normal cells. Finally, the artificial antibody was successfully used for targeting and imaging of mimetic CTCs. The present study provides a facile and efficient fluorescence tool for targeting and imaging of cancer cells.

## Results and Discussion

### Preparation and Characterization of MIP-based artificial antibody

To fabricate the glycoprotein-imprinted fluorescent artificial antibody, rhodamine doped silica nanoparticles were selected as fluorescence reporter due to its several advantages, such as better biocompatibility and ease in grafting compared with fluorescent molecules. The fluorescent silica nanoparticles were prepared through sol-gel of TEOS and APTES in the presence of rhodamine. The as-prepared fluorescent nanoparticles have several advantages, including facile modification and good water dispersity. The general scheme for the synthesis of the artificial antibody is illustrated in Fig. 1. The phenylboronic acid functionalized triethoxysilane was used to modify onto the surface of the nanoparticles. Then, template glycoprotein was immobilized onto the surface of the nanoparticles via the boronate affinity. The thickness of the imprinted layer is critical for protein recognition. Thus, the thickness of the MIPs layer was optimized by changing concentration of monomer. Therefore, the TRA-imprinted 3D cavities were created after remove of the target glycoprotein. Notably, the as-prepared MIPs-based artificial antibody has the ability to selective recognition of target TRA.

**Figure 2:**
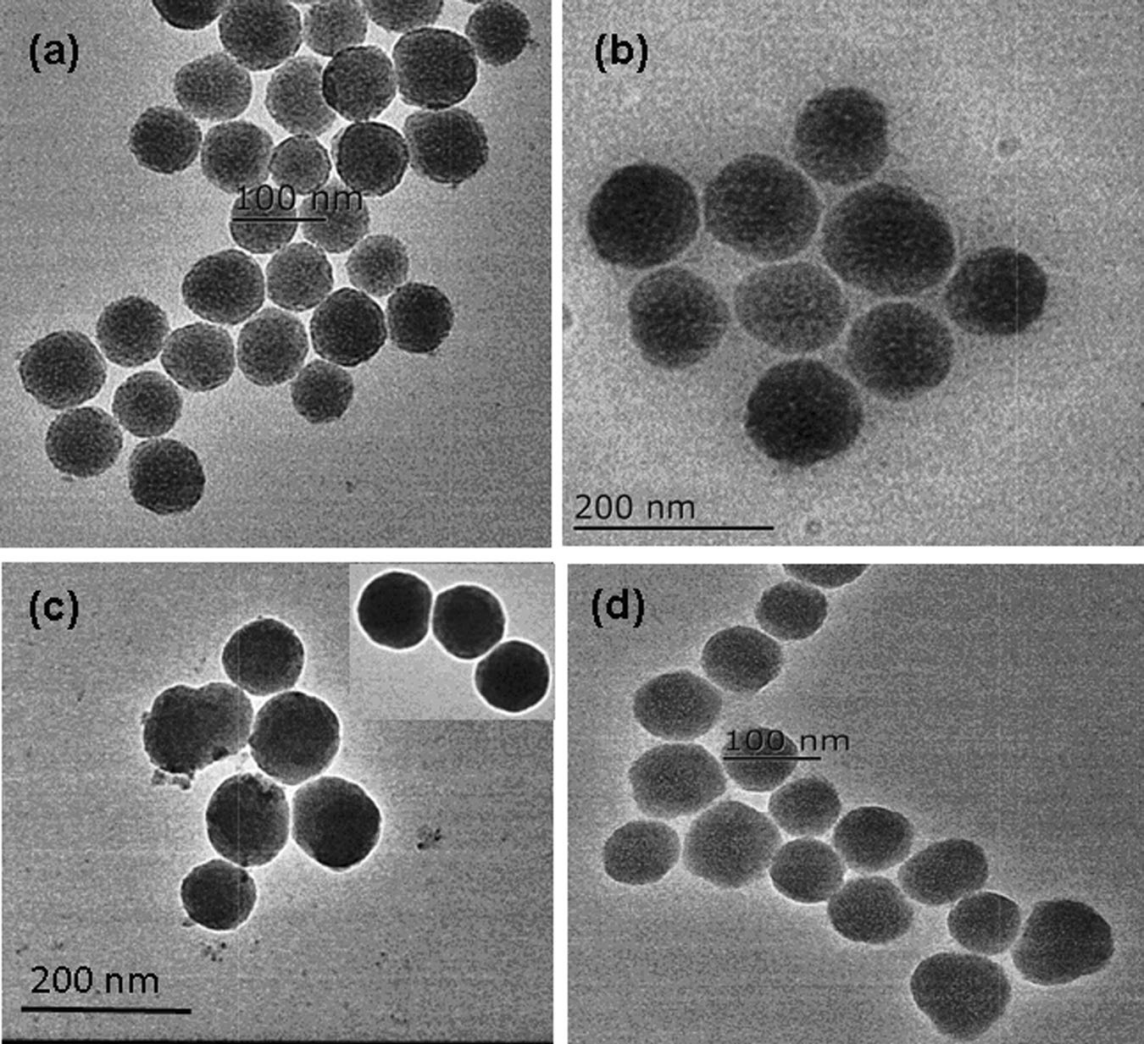
TEM images of the synthesized (**a**) rhodamine doped silica nanoparticles, (**b**) grafted with phenylboronic acid appended triethoxysilane, (**c**) MIPs-based artificial antibody, (**d**) NIP. Scale bar: a 100 nm; b 200 nm; c 200 nm; d 100 nm.

Transmission electron microscopy (TEM) images were employed to exhibit the size and shape of the nanoparticles. It can seen from Fig. 2 that the rhodamine doped silica nanoparticles had a uniform size of about 60 nm (Fig. 2a). After modified with phenylboronic acid functionalized triethoxysilane, the size of the silica nanoparticles increased (Fig. 2b). After coating with imprinted shell layer, the size and shape of the nano artificial antibody was not distinctly different from that of the non-imprinted polymer (NIP). Therefore, the different recognition performance between the MIP-based artificial antibody and the NIP in the subsequent study was attributed to the imprinting effect, but not because of the morphological difference between the MIP-based artificial antibody and the NIP. X-ray photoelectron spectroscopy (XPS) was used to investigate the elements composition of nanoparticles (Figure S1). It can be that the relative content of C 1 s and O 1 s was higher than that of the B 1 s due to the carbon and oxygen elements as the primary component in SiO_2_. The results of the XPS indicated that the functional group boric acid was grafted on the surface of the nanoparticles. The effect of pH on the fluorescence of the nano artificial antibody was evaluated (Figure S2). It can be seen that the MIP exhibit good performance at around pH 8.0 due to the boric acid exhibited good recognition ability to cis-diol under alkaline conditions, which further proved the functional group boric acid was successfully modified onto the surface of the nanoparticles.

### Recognition performance of the TRA-imprinted artificial antibody

**Figure 3:**
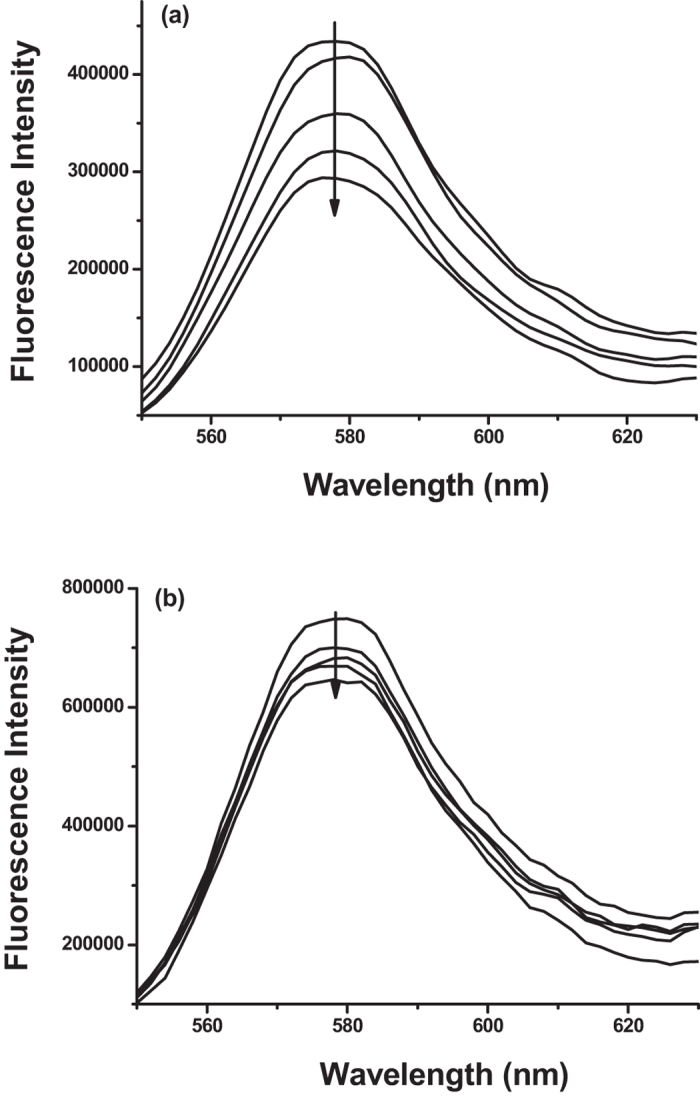
Fluorescence emission spectra of (**a**) TRA-imprinted artificial antibody and (**b**) NIP with addition of indicated concentration of target glycoprotein TRA solution. The concentration of the TRA was 1.0 ng/mL, 3.0 ng/mL, 5.0 ng/mL, 7.0 ng/mL, respectively. C_MIPs_ = C_NIP_ = 10 μg/mL.

The recognition performance of the TRA-imprinted artificial antibody was investigated according to changes of the fluorescence intensity (Fig. 3). As shown in Fig. 3, the fluorescence intensity of the TRA-imprinted artificial antibody was quenched gradually with the increase of TRA concentration, which mainly owing to the specific adsorptive affinity interaction between the artificial antibody and the target TRA. The 3D imprinted cavities and the spatial orientation of the functional sites were created in the process of imprinting. For the TRA-imprinted artificial antibody, the fluorescence quenching was mainly due to the specific affinity interaction of the 3D imprinted cavities with the target TRA, and the photo-induced electron transfer process was occurred since the distance close between the TRA and rhodamine doped in silica nanoparticles^47, 48^. Under the same conditions, the fluorescence response of the MIPs-based artificial antibody for target TRA was larger than that of the NIP, which indicated the specific recognition ability between TRA-imprinted artificial antibody and the target TRA. The generality of the method was also demonstrated by the successful imprinting of other template HRP, and the results of the HRP-imprinted artificial antibody for the template was shown in Figure S3, it can be seen that the fluorescence response of the HRP-imprinted artificial antibody for target HRP was larger than that of the NIP, which exhibited the specific recognition ability of the HRP-imprinted artificial antibody for the target HRP.

### Specificity of the MIP-based artificial antibody

**Figure 4:**
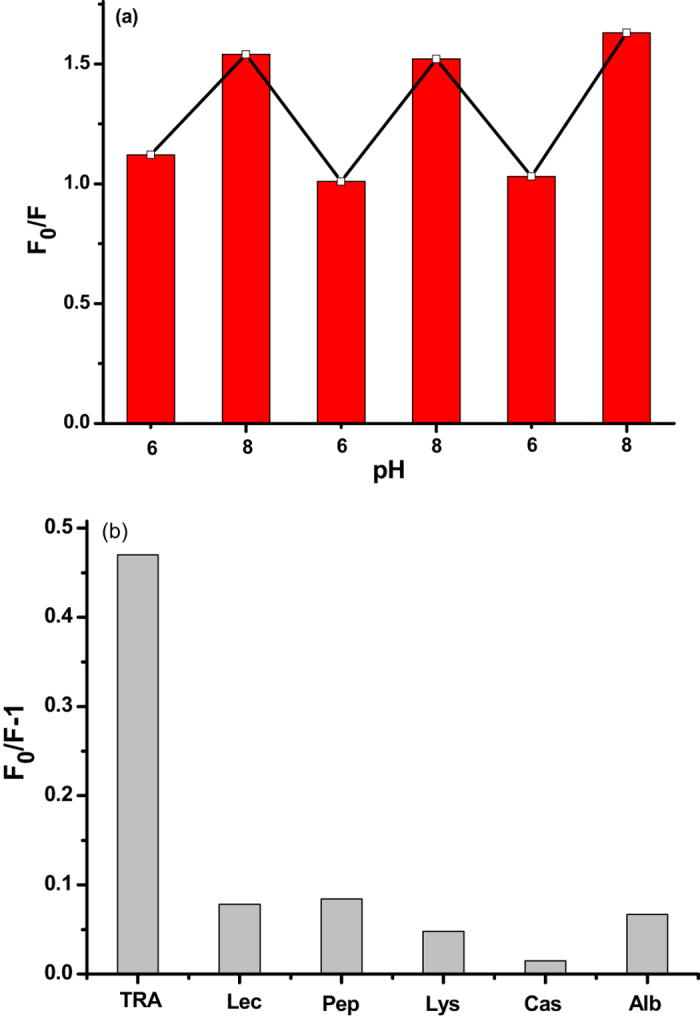
(**a**) Changes in the fluorescence intensity of the TRA-imprinted artificial antibody in the presence of the glycoprotein TRA at pH 6.0 and 8.0; (**b**) Binding behaviors of TRA-imprinted artificial antibody with target glycoprotein TRA and competitive proteins. Experimental conditions: C_MIPs_ = 10 μg/mL, C_TRA_ = C_Lec_ = C_Pep_ = C_Lys_ = C_Cas_ = C_Alb_ = 10 ng/mL.

The pH value play key role for the interaction of boronic acids with cis-diol containing compounds. Thus, the effect of the pH value on recognition performance of the artificial antibody was examined (Fig. 4). Figure 4a showed the specific binding ability of the TRA-imprinted artificial antibody for TRA at pH 6.0 and 8.0, and the artificial antibody exhibited a larger fluorescence response at pH 8.0 than that of pH 6.0. Then, the specificity of the artificial antibody was further investigated. A series of competitive protein solutions were performed to exhibit the specific recognition ability of the artificial antibody (Figs 4 and S4). It can be seen that the artificial antibody exhibited specific affinity adsorption of the target glycoprotein in the presence of structurally related proteins, which clearly demonstrated the good selective recognition ability of the artificial antibody for the target glycoprotein. Those results also demonstrated that molecularly imprinted cavities discriminated proteins on the basis of molecular shape rather than size.

### Specific binding of TRAR in cells

**Figure 5:**
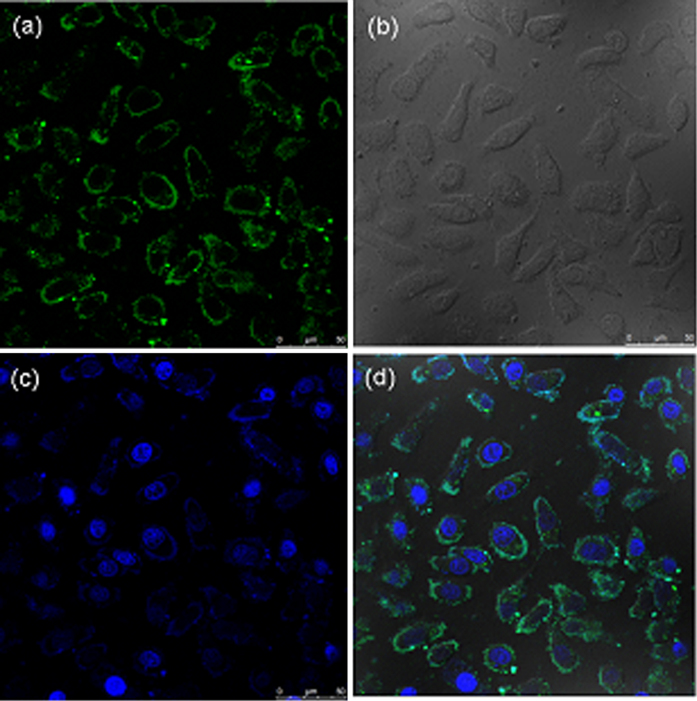
The fluorescence images of TRA-imprinted artificial antibody with TRAR in HepG 2 cells. (**a**) artificial antibody-loaded cells; (**b**) bright field confocal microscopy images of the cell; (**c**) nuclear staining with Hoechst; (**d**) merged image of (**a**,**c**). The concentration of the artificial antibody is 10 μg/mL.

**Figure 6:**
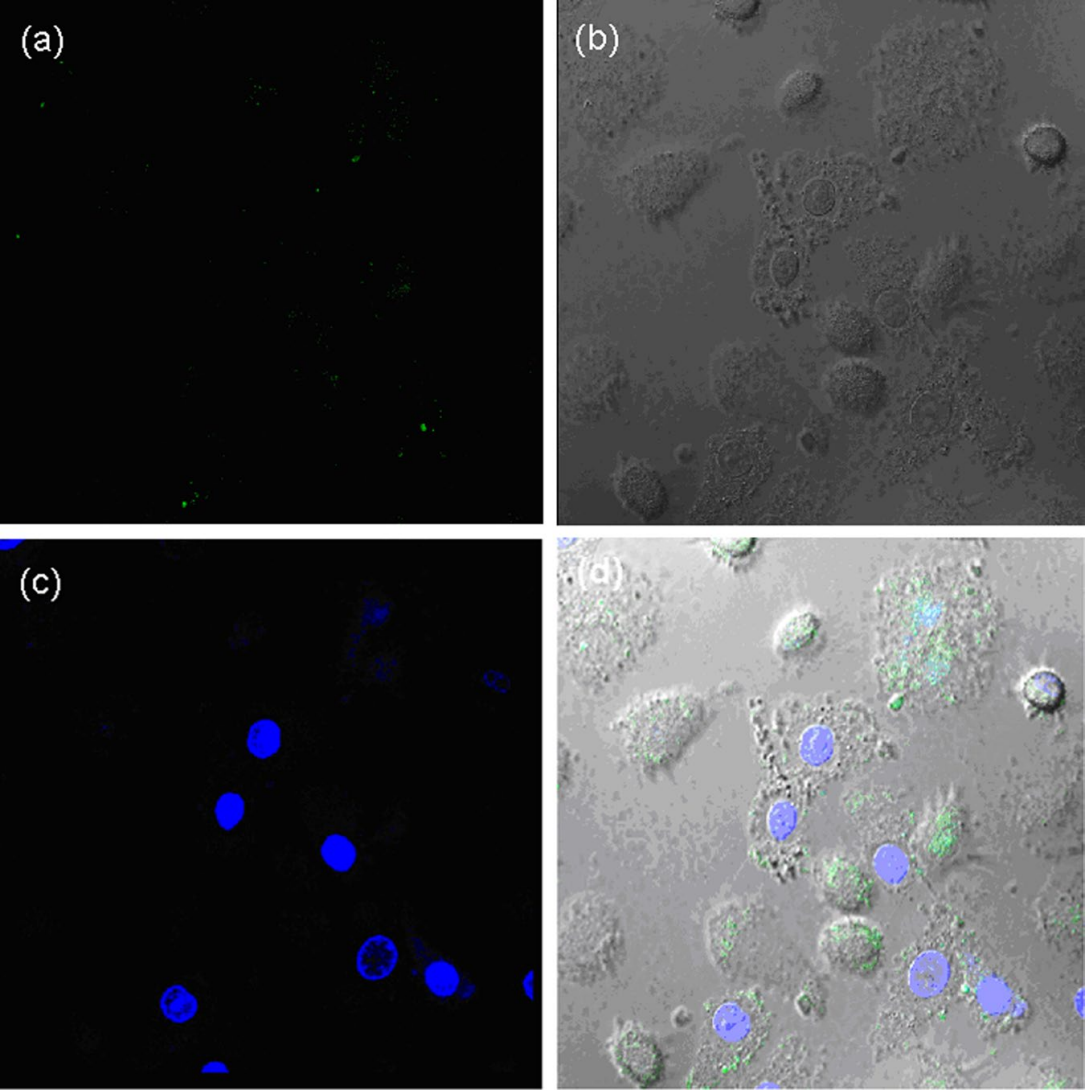
The fluorescence images of TRAR in HepG 2 cells with NIP nanoparticles mediated by TRA. (**a**) nanoparticles-loaded cells; (**b**) bright field confocal microscopy images of the cell; (**c**) nuclear staining with Hoechst; (**d**) merged image of (**a**,**c**). The concentration of the nanoparticles is 10 μg/mL.

The ability of artificial antibody to fluorescence recognition of TRAR in living cells was investigated (Fig. 5). The artificial antibody was used for specific binding with TRAR on HepG 2 cells, which known as overexpressed TRAR on HepG 2 cells membrane. As shown in Fig. 5, most of the artificial antibody appears bound on the HepG 2 cells surface, forming a ring-shaped fluorescence pattern, which indicated that the artificial antibody could fluorescence recognition of TRAR in cells. To further prove the fluorescence imaging is indeed due to the specific recognition between artificial antibody and TRAR, the NIP was used for comparison. It can be seen that the brightness of fluorescence imaging of the Fig. 6 was obviously weaker than that of Fig. 5, mainly because of less specific recognition site on the surface of the NIP. Then the hepatocyte was used to further prove the specific recognition between the artificial antibody and TRAR. Much weaker fluorescence was observed (Figure S5) due to less TRAR expressed on the normal cells, so less MIPs-based artificial antibody was marked on normal cells surface. In addition, MTT [3-(4,5-dimethylthiazol-2-yl)-2,5-diphenyltetrazolium bromide] assay in HepG2 cells was employed (Figure S6) and the results showed less cytotoxicity of the MIPs-based artificial antibody.

### Recognition CTCs in mimetic CTCs model

**Figure 7:**
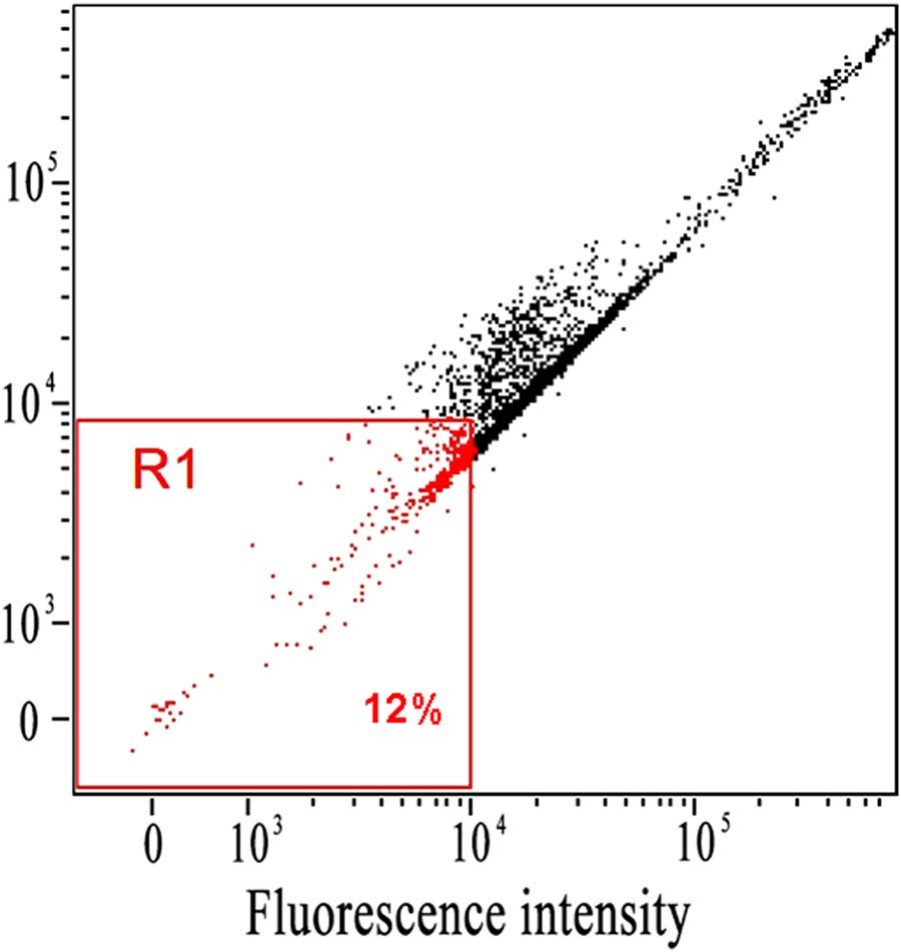
Flow cytometry characterization of recognition efficiency between the artificial antibody and HepG2. R1 represent the 12% cells were not labeled by the MIPs-based artificial antibody.

**Figure 8:**
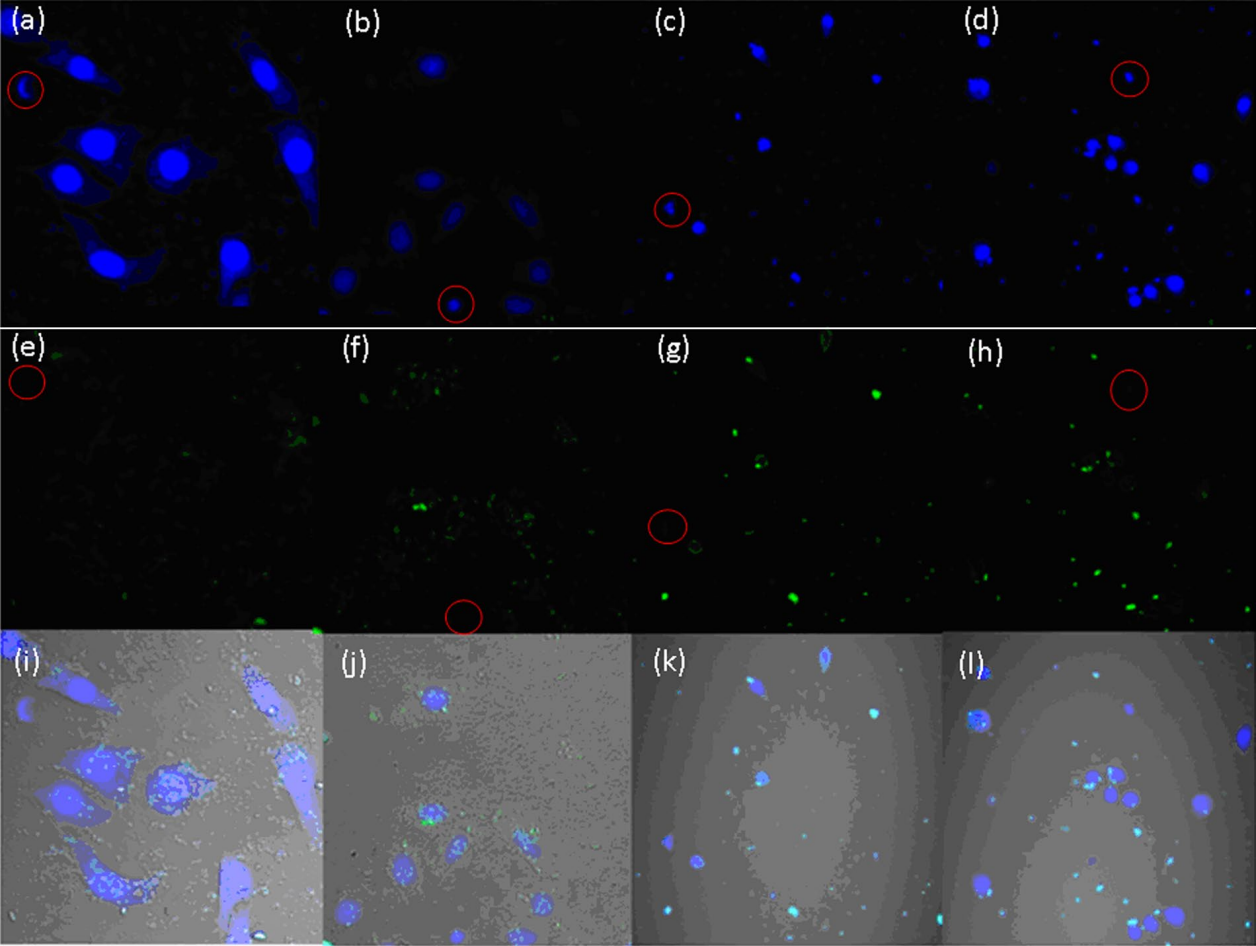
The fluorescence images of TRA-imprinted artificial antibody for targeting and labeling the CTCs in mimetic CTCs model. (**a**–**d**) nuclear staining with Hoechst, respectively; (**e**–**h**) artificial antibody-loaded cells, respectively; (**i**–**l**) merged image of (**a**,**e**,**b**,**f**,**c**,**g**,**d**,**h**), respectively. Four sets of parallel experiments were employed. The white blood cells are marked in red circles. The concentration of the artificial antibody is 10 μg/mL.

The specific recognition efficiency of the TRA-imprinted artificial antibody for the MCF-7 cells was evaluated (Figures S7–S9). It can be seen that most of the cancer cells were labeled by the artificial antibody counting by fluorescence imaging of cancer cells, and the recognition efficiency was 87%, 85% and 100%, respectively. So the average recognition efficiency was 90.6%. The flow cytometry was further used to exhibit the specific recognition between the artificial antibody and the cancer cells, it can be seen from Fig. 7 that 12% cells was not labeled by the MIP-based artificial antibody (88% cells was labeled). Notably, the recognition ability of the artificial antibody for cancer cells was further verified by natural antibody-based nanoprobe (natural antibody was used for comparison, Figures S10–S13)^48^, and the specific recognition efficiency of the TRA-imprinted artificial antibody and natural antibody-based nanoprobe for MCF-7 cells was 90.6% and 98.3%, respectively, which further showed the specific recognition ability of the artificial antibody. Finally, the TRA-imprinted artificial antibody was applied for imaging and labeling the CTCs in the mimetic CTCs model (Fig. 8). The CTCs were targeted and labeled by *in vitro* methods, and the mimetic CTCs model was established using the blood (from the tail of health mouse) and HepG 2 cells^48^. As shown in Fig. 8, the number of CTCs targeted and labeled by TRA-imprinted artificial antibody was much more than that of the white blood cells. And the data output was shown in Figure S14, it can be seen that the number of CTCs was 8 times higher than that of the white blood cells, which intuitively indicated the specific recognition ability of the TRA-imprinted artificial antibody for the CTCs. In addition, the chemical stability and recycling times of the artificial antibody was evaluated (Figures S15 and S16), the artificial antibody still exhibited good recognition ability after the artificial antibody treatment with the acid and alkali and high temperature, respectively. Compared with natural antibody-based analytical methods, the MIPs-based artificial antibody is still in infancy. However, the potential advantage of this approach in terms of simple preparation, high stability and low cost will attract more and more investigators for its wide application in future.

## Conclusion

In summary, we designed and fabricated glycoprotein-imprinted nano artificial antibody, which exhibit specific recognition ability to the target glycoprotein, and have been further applied for cell recognition mediated by target TRA. Notably, the artificial antibody as a fluorescent imaging tool exhibits desirable binding ability for TRA and could specifically recognition of cancer cells over normal cells. And the average recognition efficiency for cancer cells was 88% through flow cytometry, which further exhibited the specific recognition ability for cancer cells. Finally, the artificial antibody was successfully employed to specifically target CTCs in mimetic model. We believe that the strategy for construction of the artificial antibody will be broadly applied for monitoring and fluorescence imaging of other disease markers in biological systems.

## Methods

### Materials and Reagents

All chemicals were available commercially and the solvents were purified by conventional methods before use. Rhodamine B was purchased from Shanghai Macklin Biochemical Co., Ltd. Tetraethyl orthosilicate (TEOS), (3-Aminopropy) triethoxysilane (APTES) and 3-(Triethoxysilyl) propylisocyanate were purchased from Aladdin Industrial. Bovine Hemoglobins (BHb), Lecithos (Lec), Lysozyme (Lys), Pepsin (Pep), Horseradish Peroxidase (HRP), Casein (Cas), Transferrin (TRA), Ovalbumin (Alb) were purchased from Shanghai Lanji biological technology Co., Ltd. 3-Isocyanatopropyltriethoxysilane was purchased from Nanjing Jingruijiu biological technology Co., Ltd.

### Instruments

^1^H NMR spectra were recorded with Bruker NMR spectrometers at 300 MHz and JOEL JNM-ECA600.The mass spectra were obtained by Bruker maXis ultra high resolution TOF MS system. The fluorescence spectra measurements were performed using FLS-920 Edinburgh fluorescence spectrometer. The confocal fluorescence images were measured on a Leica TCS SP5, confocal lasers canning microscope with an objective lens (×40). The excitation wave-length was 405 nm (5 mW). UV/Vis spectra were recorded on TU-1900 UV/Vis spectrometer.

### Synthesis of Phenylboronic Acid Appended Triethoxysilane (PAAT)

The 3-aminophenylboronic acid monohydrate (80 mg, 0.5 mmol) was dissolved in THF (3 mL), then 3-isocyanatopropyltriethoxysilane (120 μL, 0.5 mmol) was added. The above-mixture was stirred at room temperature for 24 h. The formation of phenylboronic acid appended triethoxysilane (PAAT) was confirmed by MS^22^.

### Synthesis of SiO_2_@PAAT

In brief, 6 mL of TEOS was added to the mixture of 100 mL of ethanol, 4 mL of deionized water, 30 mg Rhodamine B and 3.2 mL of aqueous solution of 25% ammonium with vigorous stirring at 30 °C, the reaction was continued for 24 h and obtained the rhodamine doped silica nanoparticles. Preparation of PAAT-functionalized silica nanoparticles: To the resultant suspension, 24 mL THF of PAAT was added by stirring the mixture for 24 h with aim to obtain boric acid coated silica nanoparticles. The resultant PAAT-coated silica nanoparticles (denoted as SiO_2_@PAAT) were purified by centrifugation and washed with ethanol and deionized water, respectively. Finally, the as-prepared SiO_2_@PAAT was dried at room temperature under vacuum for further use.

### Preparation of MIP-based artificial antibody

TRA (10 mg), SiO_2_@PAAT (20 mg) was added to 10 mL PBS (30 mM, pH 8.5), and the solution was shaken for self-assembly at room temperature for 2 h. Then addition of 200 mL ethanol, 20 μL APTES, the mixed solution was stirred for 8 h at room temperature. The obtained TRA-imprinted artificial antibody was collected by centrifugation, then washed repeatedly with the solution containing SDS (10%, w/v) and acetic acid (10%, v/v), ethanol and deionized water to remove the embedded template until no TRA in the supernatant was detected using a UV/vis spectrophotometer at 280 nm. Finally, the TRA-imprinted artificial antibody was dried at room temperature for further use. The non-imprinted polymer (NIP) was prepared in the absence of template glycoprotein. In parallel, the NIP was washed with SDS, acetic acids and deionized water, respectively.

### MTT ([3-(4,5-dimethylthiazol-2-yl)-2,5-diphenyltetrazolium bromide]) Assay

HepG 2 cells (10^6^ cell mL^−1^) were dispersed within replicate 96-well microtiter plates to a total volume of 200 μL well^−1^. Plates were maintained at 37 °C in a 5% CO_2_/95% air incubator for 24 h. Then HepG 2 cells were incubated for 12 h upon different probe concentrations of 10^−5^, 10^−4^, 10^−3^, 10^−2^, and 10^−1^ mg/mL (the solvent was the TRF of the concentration of 10^−2^ mg/mL). MTT solution (5 mg/mL, PBS) was then added to each well. After 4 h, the remaining MTT solution was removed, and 150 μL of DMSO was added to each well to dissolve the formazan crystals. Absorbance was measured at 490 nm in a triturus microplate reader.

### Cell culture

HepG 2 cells and MCF-7 cells were cultured in DMEM containing 10% fetal bovine serum, 1% penicillin, and 1% streptomycin at 37 °C (w/v) in a 5% CO_2_/95% air incubator MCO-15AC (Sanyo, Tokyo, Japan). The concentrations of counted cells were adjusted to 1 × 10^6^ cells mL^−1^ for confocal imaging in high-glucose DMEM (4.5 g of glucose/L) supplemented with 10% fetal bovine serum (FBS), NaHCO_3_ (2 ng/L), and 1% antibiotics (penicillin/streptomycin, 100 U/mL). Cultures were maintained at 37 °C under a humidified atmosphere containing 5% CO_2_.

### Mimetic CTCs model

The blood^46^ was from the tail of the health mouse. Then added the blood (diluted 10 times) to the HepG 2 cells and obtained the mimetic CTCs model. The CTCs were captured from the vessel of a mouse model. Then, the CTCs were fixed with paraformaldehyde (4 wt%) for 15 min, 0.5% PBS Triton X-100 for 30 min, TRA and TRA-imprinted artificial antibody for 4 h. Then, the CTCs were identified through the fluorescence images from the artificial antibody. The mice were obtained from Shandong University Laboratory Animal Center. The experiments were approved by the Ethical Committee of Shandong University. All the animal experiments were carried out in accordance with the relevant laws and guidelines issued by the Ethical Committee of Shandong University.

### Flow Cytometry

For flow cytometry assay, HepG 2 cells were incubated for 4 h upon probe concentrations 10^−2^ mg/mL (the solvent was the TRA of the concentration of 10^−2^ mg/mL). The above prepared cells were digested with parenzyme cell digestion solution for 2–3 min. The obtained cells were centrifuged at 800 rpm for 5 min. After removing the supernatant, the cells were washed with PBS for three times. The obtained cell suspensions were injected into cytoanalyzer and the count of cells was set to 5,000.

## Electronic supplementary material

Supplementary information
