# Supplementary material for: Transferrin-navigation Nano Artificial Antibody Fluorescence Recognition of Circulating Tumor Cells
Source: Sci Rep. 2017 Aug 31;7:10142. doi: 10.1038/s41598-017-10486-9 (PMC5579199; doi:10.1038/s41598-017-10486-9)
Supplement: Supplementary file 1 — Supplementary information [file 41598_2017_10486_MOESM1_ESM.pdf]

Supplementary Information for

Transferrin-navigation Nano Artificial Antibody Fluorescence Recognition of Circulating Tumor Cells

Wei Zhang[1], Jiaoyang Wang[1], Ping Li[1], Chuanchen Wu[1], Hongyan Zhang[2], Wen Zhang[1], Hui Wang[1] & Bo Tang[1]

[1]College of Chemistry, Chemical Engineering and Materials Science, Collaborative Innovation Center of Functionalized Probes for Chemical Imaging in Universities of Shandong, Key Laboratory of Molecular and Nano Probes, Ministry of Education, Institute of Biomedical Sciences. [2]College of Life Science, Shandong Normal University, Jinan 250014, P. R. China.

*To whom correspondence should be addressed. Tel.: (86) 531-86180010; Fax: (86) 531-86180017

E-mail: tangb@sdnu.edu.cn.

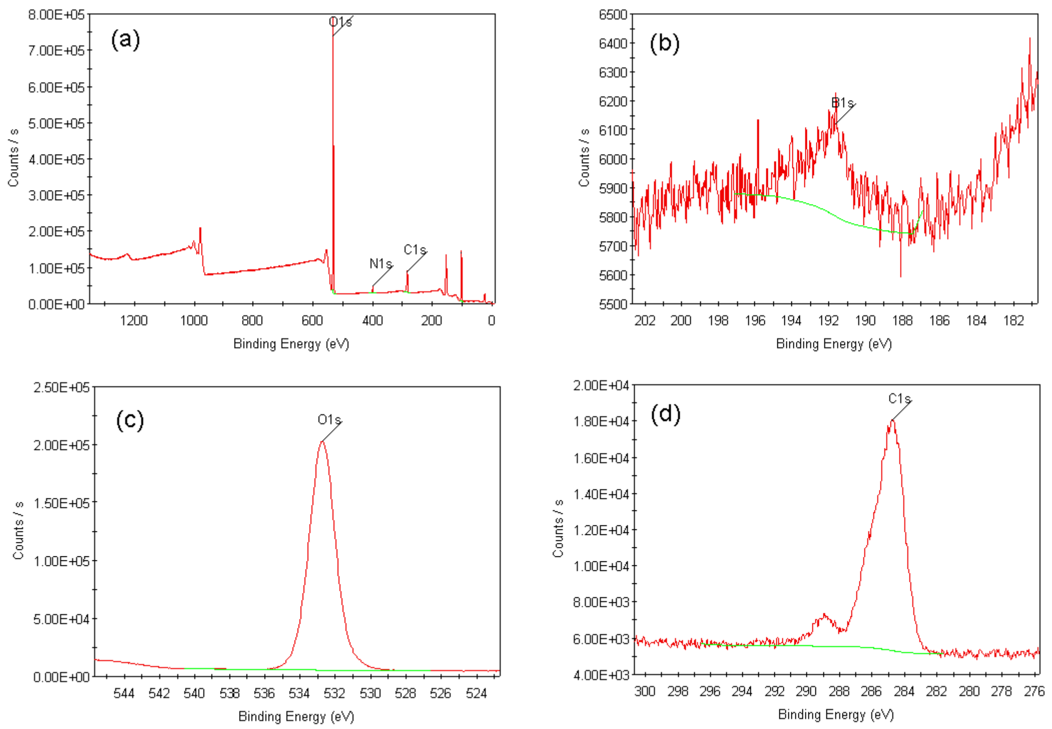

Figure S1. XPS survey (a), fitted B1s (b), O1s (c), and C1s (d) spectra of the MIP-based nanoparticles.

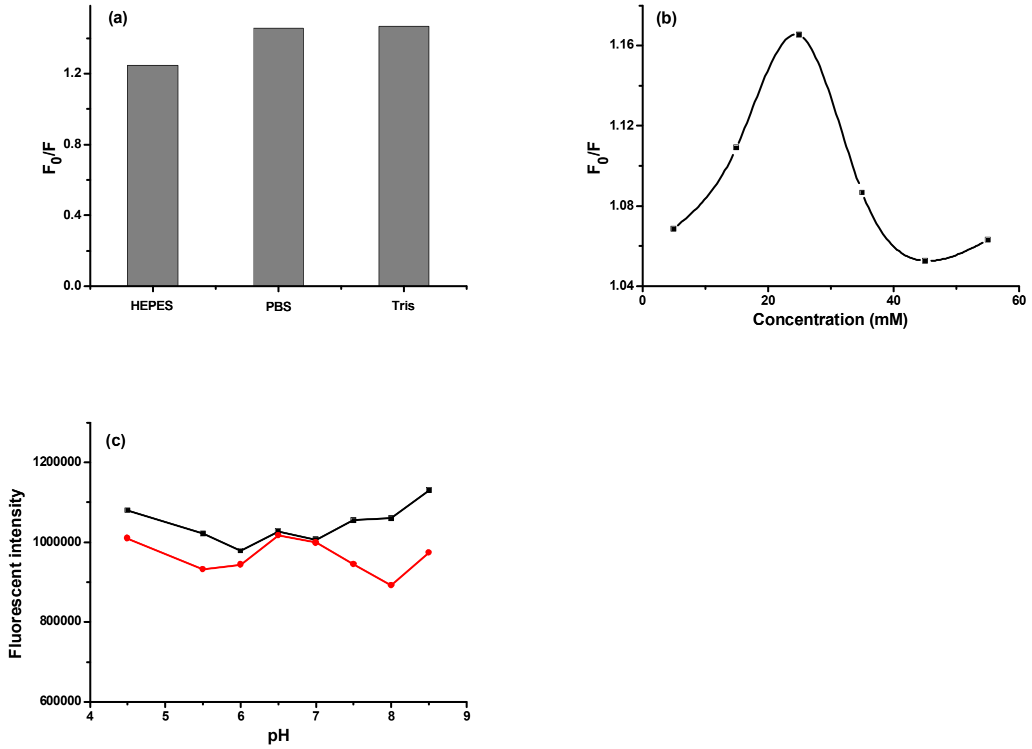

Figure S2. Conditions optimization experiment. (a) The effect of different kinds of buffer solution. (b) The effect of buffer solution concentration. (c) Effect of pH.

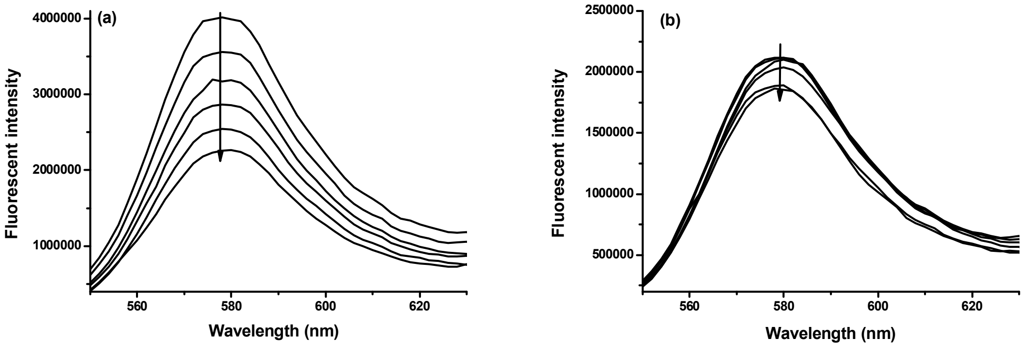

Figure S3. Fluorescence emission spectra of (a) HRP-imprinted artificial antibody and (b) NIP with addition of indicated concentration of target glycoprotein HRP solution. The concentration of the HRP was 1.0 ng/mL, 3.0 ng/mL, 7.0 ng/mL, 8.0 ng/mL, 10.0 ng/mL, respectively. $C_{MIPs} = C_{NIP} = 10$ μg/mL.

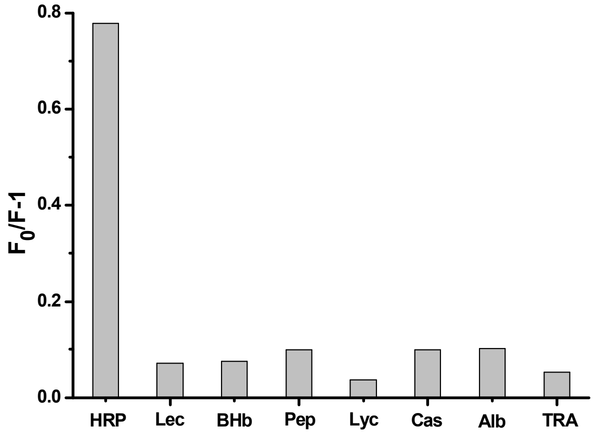

Figure S4. Binding behaviors of target glycoprotein HRP and competitive proteins on the HRP-imprinted artificial antibody. Experimental conditions: C_{MIPs}= 10 μg/mL, $C_{HRP} = C_{Lec} = C_{BHb} = C_{Pep} = C_{Lys} = C_{Cas} = C_{Alb} = C_{TRA}$ =10 ng/mL.

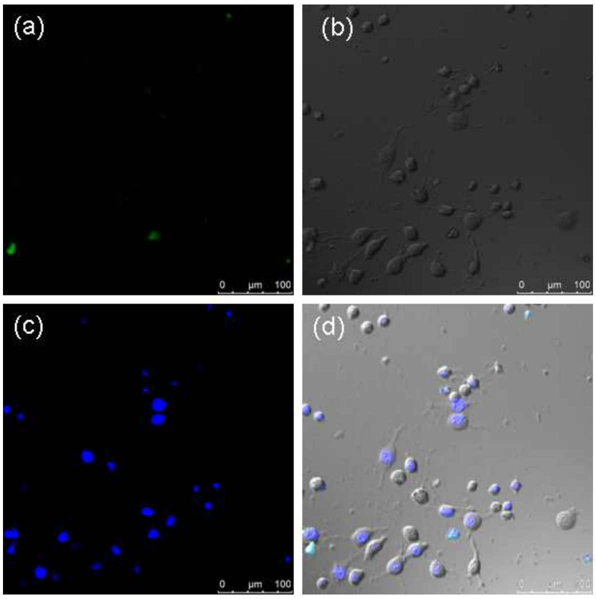

Figure S5. The fluorescence images of TRAR in hepatocyte cells with TRA-imprinted artificial antibody. (a) artificial antibody-loaded cells; (b) bright field confocal microscopy images of the cell; (c) nuclear staining with Hoechst (d) merged image of (a) and (c). The concentration of the artificial antibody is 10 μg/mL.

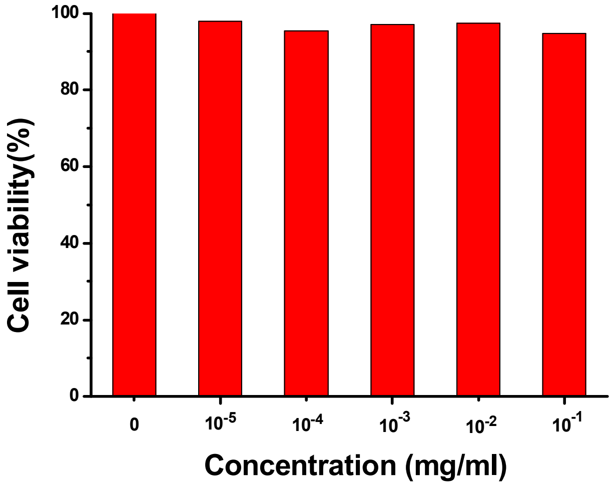

Figure S6. Viability of HepG 2 cells in the presence of TRA-imprinted artificial antibody as measured by using MTT assay. The cells were incubated with artificial antibody for 12 h.

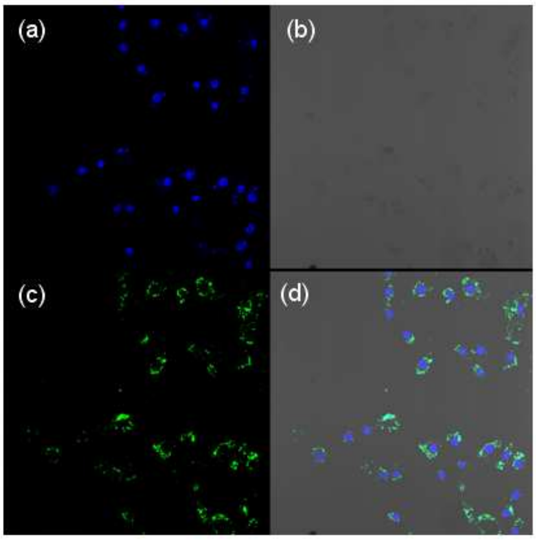

Figure S7. The recognition efficiency of the TRA-imprinted artificial antibody for the MCF-7 cells. (a) nuclear staining with Hoechst; (b) bright field confocal microscopy images of the cell; (c) artificial antibody-loaded cells; (d) merged image of (a) and (c). (a: total of 38 cells; b: able to identify 33 cells; the recognition efficiency was 87%). The concentration of the artificial antibody is 10 μg/mL.

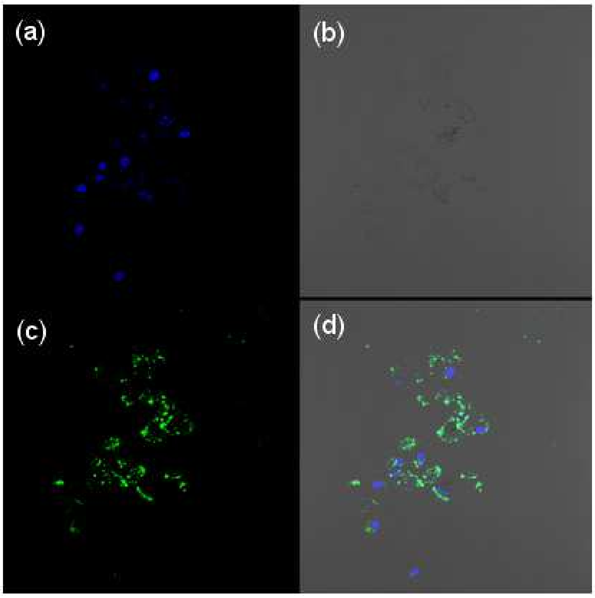

Figure S8. The recognition efficiency of the TRA-imprinted artificial antibody for the MCF-7 cells. (a) nuclear staining with Hoechst; (b) bright field confocal microscopy images of the cell; (c) artificial antibody-loaded cells; (d) merged image of (a) and (c). (a: total of 26 cells; b: able to identify 22 cells; the recognition efficiency was 85%). The concentration of the artificial antibody is 10 μg/mL.

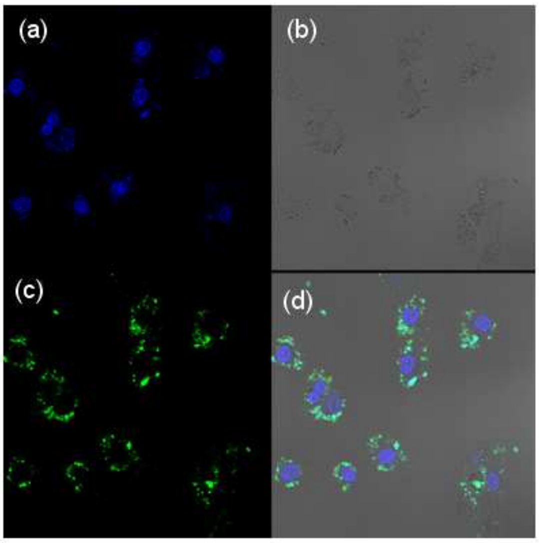

Figure S9. The recognition efficiency of the TRA-imprinted artificial antibody for the MCF-7 cells. (a) nuclear staining with Hoechst; (b) bright field confocal microscopy images of the cell; (c) artificial antibody-loaded cells; (d) merged image of (a) and (c). (a: total of 13 cells; b: able to identify 13 cells; the recognition efficiency was 100%). The concentration of the artificial antibody is 10 μg/mL.

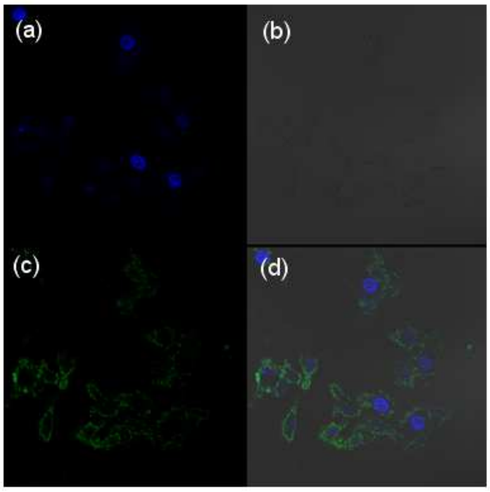

Figure S10. The recognition efficiency of the natural antibody-based nanoprobe for the MCF-7 cells. (a) nuclear staining with Hoechst; (b) bright field confocal microscopy images of the cell; (c) natural antibody-based nanoprobe-loaded cells; (d) merged image of (a) and (c). (a: total of 17 cells; b: able to identify 17 cells; the recognition efficiency was 100%). The concentration of the natural antibody-based nanoprobe is 10 μg/mL.

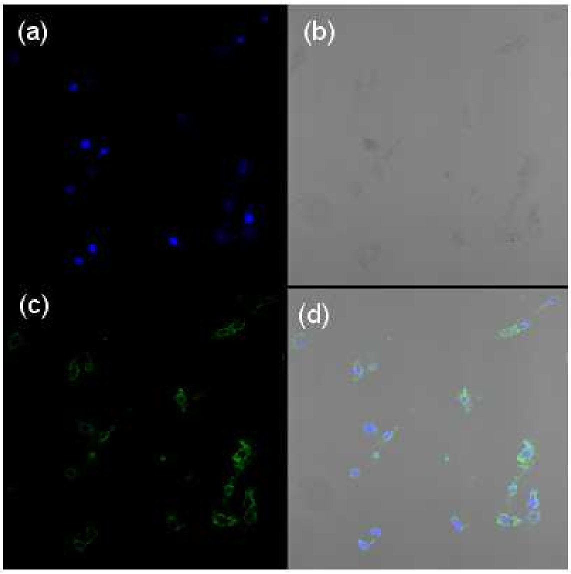

Figure S11. The recognition efficiency of the natural antibody-based nanoprobe for the MCF-7 cells. (a) nuclear staining with Hoechst; (b) bright field confocal microscopy images of the cell; (c) natural antibody-based nanoprobe-loaded cells; (d) merged image of (a) and (c). (a: total of 19 cells; b: able to identify 18 cells; the recognition efficiency was 95%). The concentration of the natural antibody-based nanoprobe is 10 μg/mL.

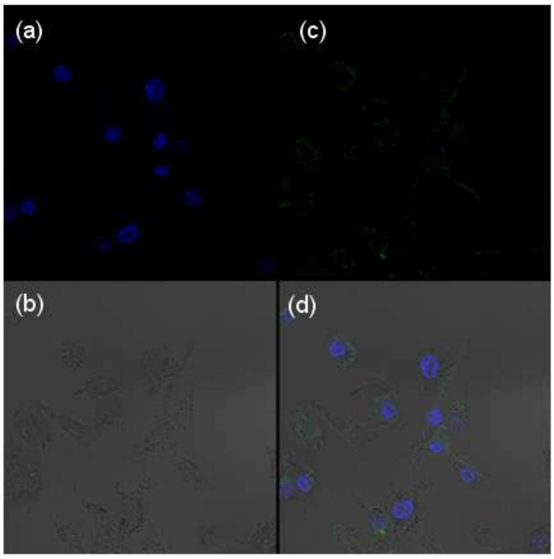

Figure S12. The recognition efficiency of the natural antibody-based nanoprobe for the MCF-7 cells. (a) nuclear staining with Hoechst; (b) bright field confocal microscopy images of the cell; (c) natural antibody-based nanoprobe-loaded cells; (d) merged image of (a) and (c). (a: total of 18 cells; b: able to identify 18 cells; the recognition efficiency was 100%). The concentration of the natural antibody-based nanoprobe is 10 μg/mL.

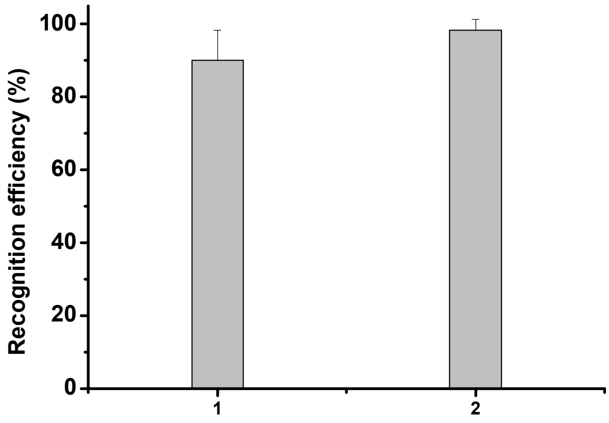

Figure S13. The recognition efficiency of the TRA-imprinted artificial antibody (1) and natural antibody-based nanoprobe (2) for the MCF-7 cells was 90.6% and 98.3%, respectively.

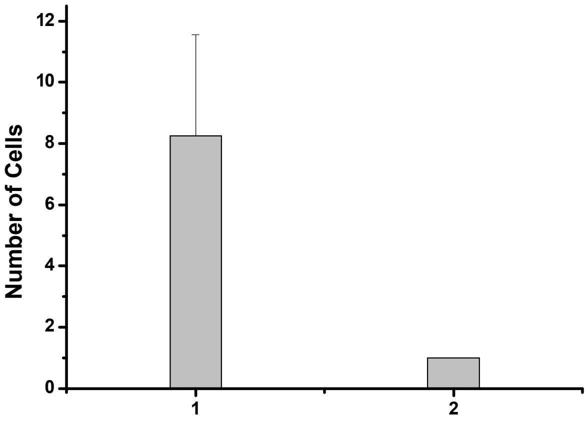

Figure S14. The data output fluorescence intensities of the TRA-imprinted artificial antibody specific binding of the CTCs and the white blood cells in Figure 6 (Column 1: the number of the CTCs; Column 2: the number of the white blood cells).

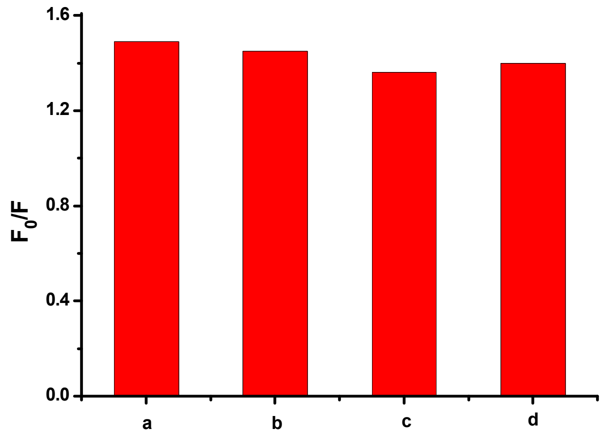

Figure S15. The chemical stability of the artificial antibody. (a) Fluorescence response of the HRP-imprinted artificial antibody with HRP; (b) The fluorescence response of the HRP with artificial antibody after the artificial antibody with acid treatment; (c) The fluorescence response of the HRP with artificial antibody after the artificial antibody with alkali treatment; (d) The fluorescence response of the HRP with artificial antibody after the artificial antibody with high temperature (100 °C) treatment. The experimental details: C_{HCl}= C_{NaOH}= 0.1 M, the high temperature is 100 degrees, the concentration of the artificial antibody is 10 μg/mL, C_{HRP}= 1.0 ng/mL.

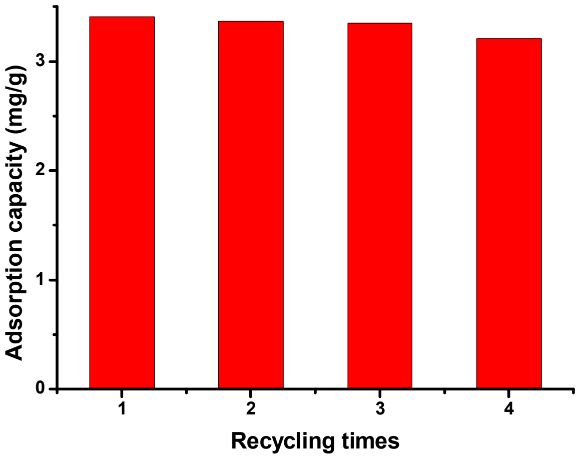

Figure S16. The recycling times of the MIPs. Experiment was conducted by the addition of 20 mg of MIPs-based artificial antibody in 0.2 mg/mL glycoprotein solution at room temperature.

The adsorption capacity (Q, expressed in units of mg/g) of the target glycoproteins bound to the MIPs is calculated by

$$Q = (C_0 - C_t)\ V / W$$

where C_0 and C_t (mg/mL) are the initial concentration and the residual concentration of the target glycoproteins, respectively, V (mL) is the volume of the initial solution, and W (g) is the weight of the MIPs.

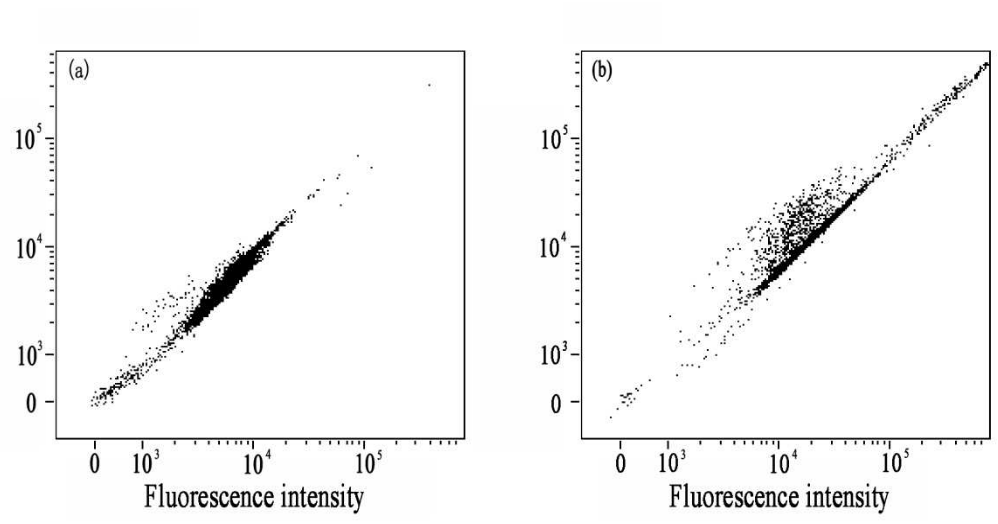

Figure S17. Flow cytometry characterization of recognition efficiency between the artificial antibody and HepG2 cells. (a) control group; (b) experimental group.

Table S1. Results for the quantitative detection of HRP in human HepG2 cell.

Sample	HRP concentration [μg/mL]		Amount detected[%][a]
	spiked	measured[a]	
1	0.0	n.d.[b]	
2	50.0	51.3±0.9	102.3±2.3
3	80.0	80.8±2.7	101.0±3.5
4	100.0	114.0±14.2	114.1±14.1
5	150.0	150.1±7.8	100.0±5.2

[a] The mean ± the standard deviation for three experiments is given.
[b] Not detected

The developed HRP-imprinted artificial antibody was employed for the selective detection of HRP in biological fluids, such as human HepG2 cell. An appropriate (100-fold) diluted solution of human HepG2 cell spiked with HRP was found to be sufficient for the quantitative recovery of HRP (Table S1).
